# Combination Prostatic Artery Embolization Prior to Water-Jet Ablation (Aquablation) for Benign Prostatic Hypertrophy: A Propensity Score Analysis

**DOI:** 10.3390/jcm13226930

**Published:** 2024-11-18

**Authors:** Sandeep Bagla, Inderjit Singh, Abin Sajan, Antony Sare, Alex Pavidapha, Tej Mehta, John Klein, Shawn Marhamati, Lori Lerner

**Affiliations:** 1 IR Centers USA, 2755 Hartland Road, Falls Church, VA 22043, USA; sbagla@prostatecentersusa.com (S.B.); apavidapha@prostatecentersusa.com (A.P.); 2 Potomac Urology, 1800 N. Beauregard Street, Alexandria, VA 22311, USA; isingh@potomacurology.com (I.S.); jklein@potomacurology.com (J.K.); smarhamati@potomacurology.com (S.M.); 3 Department of Radiology, Columbia University Irving Medical Center, 622 W 168th St, New York, NY 10032, USA; 4 Department of Radiology & Biomedical Imaging, Yale University School of Medicine, P.O. Box 208042, New Haven, CT 06520-8042, USA; antony.sare@gmail.com; 5 Department of Radiology, Johns Hopkins Hospital, 1800 Orleans St, Baltimore, MD 21287, USA; tejim@live.com; 6 Department of Surgery, Boston University Chobanian & Avedisian School of Medicine, 72 East Concord Street, Boston, MA 02118, USA; lerner.lori2014@gmail.com

**Keywords:** benign prostatic hypertrophy, aquablation, prostatic artery embolization

## Abstract

**Objectives:** To compare post-operative bleeding measures in patients who underwent prostatic artery embolization (PAE) prior to water-jet ablation (aquablation) vs. water-jet ablation alone. **Methods:** A retrospective review identified 145 patients treated with water-jet ablation for benign prostatic hyperplasia from December 2018 to June 2021. Patients were divided into two groups: water-jet ablation alone (n = 56) vs. pre-operative PAE and water-jet ablation (n = 89). Patient demographics, pertinent laboratory values, operative reports, and hospital courses were reviewed. **Results:** PAE was technically successful in all patients (n = 89), and all 89 patients underwent successful water-jet ablation within a median time of 2 days. Compared to water-jet ablation alone, pre-operative PAE resulted in a significant reduction in post-operative bleeding as measured via lower rates of continuous bladder irrigation, hemostatic measures, and hematuria. Pre-operative PAE was also associated with lower rates of post-operative urinary retention (odds ratio 17, *p* = 0.02) and less likely to require reoperation 30 days after the procedure (*p* = 0.003). There were no major PAE-related adverse events reported in the combination arm. **Conclusions:** Compared to water-jet ablation alone, pre-operative PAE resulted in fewer bleeding-related complications and urinary retention.

## 1. Introduction

Water-jet ablation has emerged as an alternative to transurethral resection of the prostate to treat bladder outlet obstruction (BOO) in the setting of benign prostatic hyperplasia (BPH) [1]. The system utilizes ultrasound guidance and robotic technology to precisely ablate prostate tissue with a high-pressure water jet. The major advantages of this procedure are the ability to treat prostates of various sizes, even those in the large and very large category, quickly and efficiently; reduced sexual side effects compared to other procedures for larger glands; and a shorter learning curve than many resection techniques [2]. However, a known disadvantage of water-jet ablation is the potential for bleeding complications largely, if not completely, attributable to the non-thermal nature of the procedure. Transfusion rates as high as 6–10% were reported in early clinical trials [3,4]. More recent approaches to minimize bleeding complications include post-operative cautery using monopolar, bipolar, or laser energy [5]. This may extend the length of the surgery and increase the risk for delayed bleeding, especially in patients with risk factors such as larger prostate sizes and those on anticoagulants.

Prostatic artery embolization (PAE) is a proven treatment for hematuria of prostatic origin that can be performed with very low risk of serious complications in an outpatient setting with moderate sedation or local anesthesia [6,7,8]. Therefore, performing a PAE prior to water-jet ablation may be a reasonable adjunct procedure if the combination reduces bleeding complications, both early and delayed. Multiple retrospective and prospective studies have identified that PAE is also effective in reducing prostate volume in patients with BPH [7,8,9,10,11,12,13]. It is similar in effectiveness to other minimally invasive surgical treatment options such as Rezum and Urolift [14,15,16]. The purpose of this analysis was to quantify the effect of pre-operative PAE in reducing post-procedural bleeding and the need for bleeding-related therapeutic measures.

## 2. Materials and Methods

### 2.1. Study Population and Experimental Design

This retrospective study was approved by an Institutional Review Board (Sterling IRB), and study activities were performed with HIPAA compliance. Records were reviewed from 145 consecutive patients who underwent water-jet ablation by a single operator from December 2018 to June 2021 for severe lower urinary tract symptoms (LUTS) secondary to benign prostatic hypertrophy.

Data collected included baseline age, pre-operative prostate volume (transrectal US), urological surgical history, pre- and post-operative assessments/medications (International Prostatic Symptom Score (IPSS), Qmax, Post-Void Residual (PVR), prostate specific antigen (PSA), quality of life (QoL), and International Index of Erectile Function (IIEF)), information related to water-jet ablation surgery, hospitalization (technical reports, operative time, duration of stay, and complications), and post-operative events (re-admission, re-intervention, etc.). If the patient received a combination treatment with pre-operative PAE, information pertaining to the PAE procedure was obtained (technical reports, technical success, complications). Prostatic volumes from standard of care transrectal ultrasound were also recorded. Adverse events were assessed according to the Society of Interventional Radiology (SIR) Classification System [17]. Operative complications were assessed according to the Clavien–Dindo Scale [18].

### 2.2. Patient Selection

The patients were selected to undergo pre-operative PAE based on the preference of the operating surgeon. The selection criteria included prostate gland size, patient comorbidities, and presence of anticoagulation.

### 2.3. Procedure

**Figure 1:**
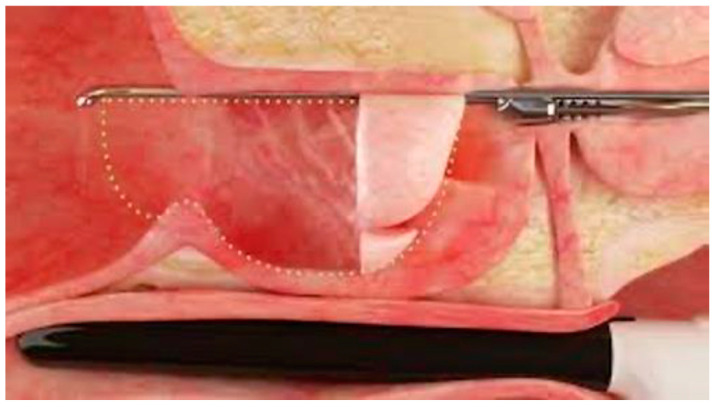
Schematic diagram demonstrating water-jet ablation of prostatic tissue.

Water-jet ablation: Water-jet ablation was performed using the AquaBeam Robotic System (PROCEPT BioRobotics, Redwood Shores, Redwood, CA, USA). After administering general anesthesia, the patients were positioned in the lithotomy position. Biplanar BK transrectal ultrasound was inserted and appropriately positioned in the rectum before locking into place on the robotic arm. Using a freehand approach, the cystoscope and jet apparatus were then advanced and locked into place. The ultrasound was adjusted to visualize the prostate in the sagittal plane. The depth of treatment start, bladder neck, mid-prostate, verumontanum, and distal extent of treatment were mapped. Once appropriately adjusted, water-jet ablation was performed according to the mapped plan (Figure 1). Additional passes, usually 1–3, were performed as necessary. After water-jet ablation was complete, hemostasis was achieved with a combination of loop electrode cautery and/or Foley catheter traction, as required. The use of the above hemostatic measures was compared between the two groups. All patients were then started on continuous bladder irrigation.

**Figure 2:**
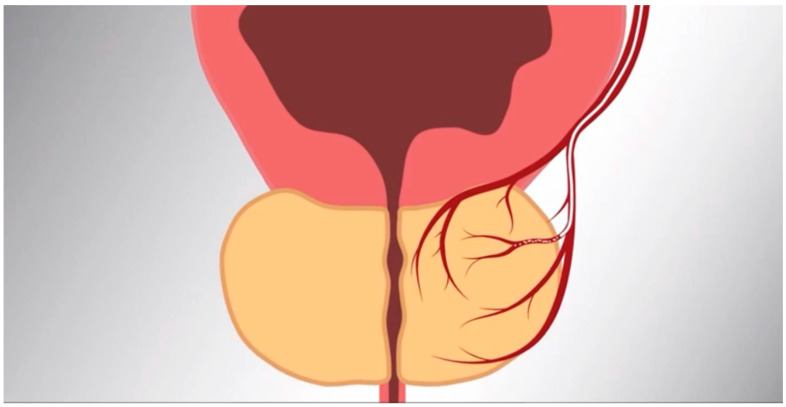
Schematic diagram with a large hypervascular prostate resulting in bladder outlet syndrome. A catheter is placed into prostatic arteries, and particles are released to decrease blood flow.

**Figure 3:**
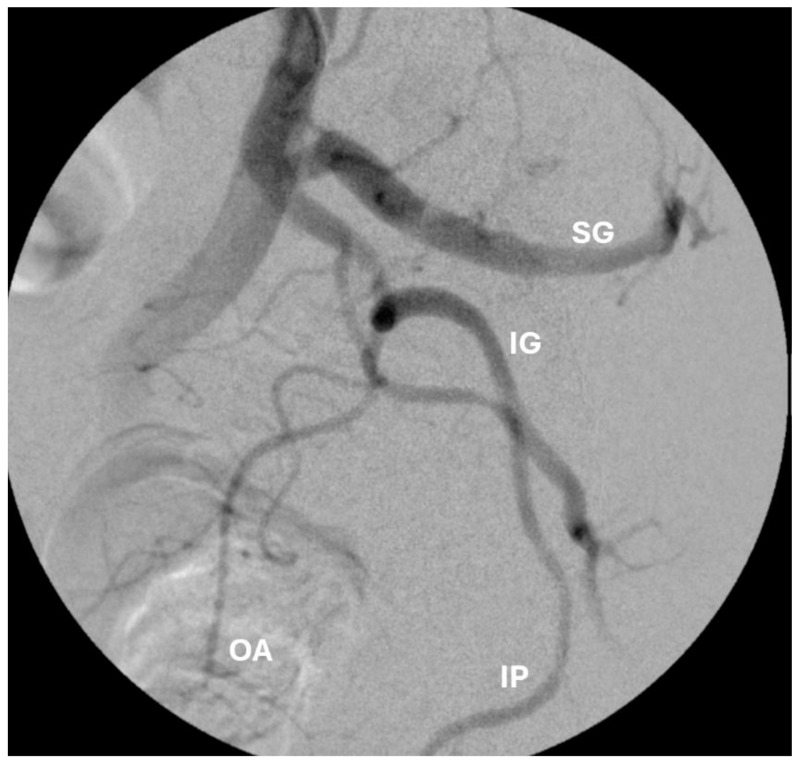
Angiogram of the internal illiac artery demonstrating the relevant prostate artery embolization anatomy. SG: superior gluteal. IG: inferior gluteal. IP: internal pudendal. OA: obturator artery.

**Figure 4:**
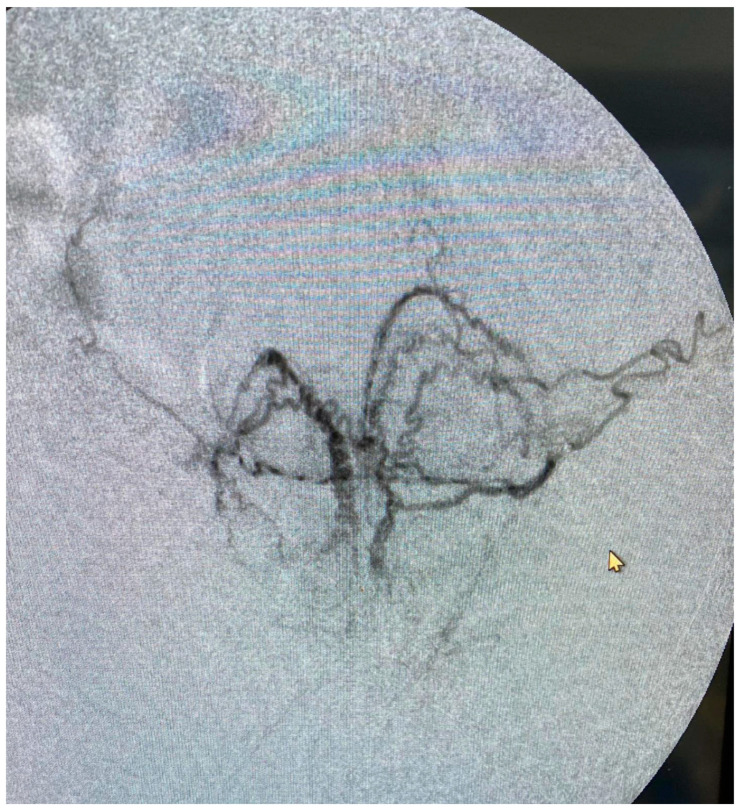
Angiogram of the distal prostatic arteries demonstrating the size and intensity of the periurethral vasculature.

Prostatic artery embolization: PAE was performed in a similar fashion as previously described [7] (Figure 2). Cone beam CT was not performed for any intra-procedural planning. If aberrant anastomoses were identified on digital subtraction angiography, coil embolization or selective prostatic artery catheterization was performed to prevent non-target embolization. Embolization was performed using small-caliber microspheres, 100–300 microns (Embozene, Boston Scientific, Marlborough, MA, USA, or Hydropearl, Terumo Medical, Somerset, NJ, USA) and gel–foam slurry, until complete stasis was achieved (Figure 3 and Figure 4). The selection between Embozene and Hydropearl microspheres was based on operator preference, and outcomes differences between the two groups were not collected.

To compare differences in baseline patient characteristics and outcomes between the two groups, Pearson’s chi-square or Fisher’s exact test were used to analyze categorical variables, and Mann–Whitney U-test or Student’s t-test were used for continuous variables.

Propensity score matching was performed to minimize the effects of hidden, non-random confounding variables and selection biases. The 1:1 nearest neighbor propensity score matching without replacement was first attempted with a propensity score estimated using logistic regression of the treatment on the covariates. This matching yielded poor balance, leading to the full matching on the propensity score, which yielded adequate balance. The propensity score was estimated using a probit regression of the treatment on the covariates, which yielded better balance than did a logistic regression. A standardized mean difference was used to evaluate the balance of the covariate distribution between the 2 groups. After matching, the marginal odds ratio was used to analyze categorical variables, while the Wilcoxon signed-rank test or paired t test were employed to compare continuous variables.

A 2-tailed *p* value of <0.05 was considered statistically significant. All statistical analyses were performed using R (version 4.04; R Foundation for Statistical Computing, Vienna, Austria). The R package MatchIt was used for propensity score matching.

## 3. Results

### Baseline Characteristics

56 patients received water-jet ablation alone, and 89 patients received pre-operative PAE. In the combined group, patients underwent water-jet ablation within 7 days of PAE. There were significant differences in baseline age and prostate gland size between the two groups (Table 1). The mean age for the water-jet ablation group was 62.2 ± 8.6 (SD) years, and the combination group was 69.6 years ± 8.9 (*p* < 0.001). Mean prostate gland size among the water-jet ablation group was 52.6 ± 24.8 mL and among the combination group was 79.0 ± 27.3 mL (*p* < 0.001). After propensity score matching, these variables were adequately balanced (Table 2). Pre-treatment variables, including PSA, Qmax, PVR, IPSS, QOL, and IIEF, were not significantly different between groups with both pre- and post-propensity score matching analyses.

There were no significant differences in length of hospital stay after propensity score matching.

In the immediate post-operative period, patients in the water-jet ablation group were 7.6 (OR) times more likely to require continuous bladder irrigation > 2 h post-operation (n = 43 vs. 30, *p* < 0.001) (Table 3). The water-jet ablation-only treatment group was also 14.7 times more likely to require hemostatic measures during their hospital stay (n = 57 vs. 45, *p* < 0.001). There was no difference in use of additional manual bladder irrigation of the Foley catheter between the water-jet ablation and combination treatment groups during their hospital stay (*p* = 0.15).

During the follow-up period (mean = 4.1 ± 3.1 months, range 0–33 months), patients that received water-jet ablation alone were 3.0 times more likely to experience prolonged hematuria (>6 weeks post-op) (n = 22 vs. 16, *p* = 0.01). Similarly significant differences were also recorded for post-operative urinary retention (n = 7 vs. 3, *p* = 0.02). There was no difference in rates of post-operative dysuria between groups.

Patients in the water-jet ablation group were 13 times more likely to return to the OR due to a procedural complication after 30 days (n = 6 vs. 3, *p* = 0.003). No significant differences were demonstrated when comparing long-term Foley catheter requirements and return to OR within 30 days.

## 4. Discussion

As a newer technique to treat bladder outlet obstruction secondary to BPH, water-jet ablation has been proven to achieve a similar reduction in urinary symptoms with a lower rate of ejaculatory dysfunction when compared to TURP [19]. Unlike other minimally invasive treatments such as Rezum (Boston Scientific, Marlborough, MA, USA) and Urolift (Teleflex, Pleasanton, CA, USA), water-jet ablation can effectively treat large prostates greater than 80 mL [20]. However, one disadvantage with larger prostates is the incidence of bleeding complications compared to other surgical modalities, with post-procedural transfusion rates as high as 10% [3]. There remains some degree of uncertainty regarding how bleeding should be managed during the procedure, but a combination of both electrocautery and traction techniques has been reported to yield the best results [21].

PAE has been shown to be a successful stand-alone therapy in reducing lower urinary symptoms secondary to BPH in prostate glands of all sizes [22]. The use of pre-operative embolization to reduce intraoperative blood loss is a well-established practice, most performed prior to hypervascular tumor resection, such as renal cell carcinoma [23]. More recently, the benefit of embolization before simple prostatectomy to treat BPH has been described [24]. Pre-operative embolization has also evolved outside of urology to include practices such as head and neck tumors, fibroids, musculoskeletal lesions, and hepatic oncology [25].

The concept of utilizing pre-operative PAE has a similar rationale given the rate of bleeding complications. Potential non-target embolization remains the main disadvantage of performing PAE before water-jet ablation. However, given that PAE is associated with very few severe adverse events (less than 0.3%), the combined approach to BPH offers a potential solution to prevent bleeding-related complications [26]. The benefits are likely higher in patients with increased vascularity, such as larger glands with or without indwelling catheters, and those with recurring infections. These benefits need to be weighed against the cost and risk of two procedures, both for the patient and the healthcare system. The medical and financial implications of two procedures are balanced with potentially improved patient factors such as same-day discharge, lower rates of bleeding-related complications, faster recovery time, and quicker return to pre-procedural activities.

The two comparative groups in this report contain large sample sizes compared to other PAE and water-jet ablation analyses, lending strength to the conclusions. The difference in prostate volume between the two groups merits discussion. Patients with risk factors for bleeding, such as larger gland sizes, were referred for PAE based on the operator’s judgment. Given the known higher rate of bleeding complications after water-jet ablation in prostates greater than 80 mg, this group would likely stand to benefit from pre-operative PAE more than those with smaller prostate glands. However, propensity score matching was utilized to adjust for this difference. After this adjustment, the data demonstrated improved outcomes with combination therapy, with a lower incidence of prolonged hematuria, urinary retention, and a reduced need for post-surgical procedures to manage the hematuria.

Fassia et al. reported a similar study recently looking at 11 patients that underwent pre-aquablation PAE [27]. Unlike the current study, the prostate sizes were larger, measuring with a mean volume of 189.9 mL. This feasibility study did not have a comparison group but reported indicators of bleeding such as hemoglobin and platelet levels. Although the mean post-procedure change was 1.4 g/dL, there were no cases of bleeding complications requiring blood products.

Combination therapy for BPH is increasingly common, especially involving PAE, as the American Urologic Association guidelines on BPH recently included PAE as a possible treatment option [28,29]. Babore et al. reported on 17 patients that underwent PAE approximately 48.4 months following Urolift [30]. The degree of symptom relief was similar to a treatment-naive PAE control group at follow-up. Varadhan et al. also reported on salvage PAE in a group of 16 patients with BPH [31]. Prior minimally invasive surgical treatments included Urolift (n = 6), Rezum (n = 5), GreenLight (n = 3), transurethral needle ablation (n = 1), and transurethral microwave thermotherapy (n = 1). PAE was performed approximately 3.3 years after the initial treatment. PAE was technically feasible in all patients, and significant improvements in IPSS and QoL were reported in all patients.

The main limitations of this analysis were the retrospective nature of data collection, the lack of randomization between the comparative groups, and the differences in prostate sizes. If the data were collected prospectively in a randomized fashion, there likely would not have been a difference in prostate volume between the two groups. Additionally, there would likely be a significant difference in length of stay if the data were more homogenous. Instead, this represents a “real world” dataset in which patients with larger prostates were more likely to undergo combination therapy due to greater concern for bleeding complications. Although additional data are necessary, combination therapy in smaller glands is less efficacious, as PAE is technically challenging with increased risk and cost to the patient and to the healthcare system. Pre-operative PAE is most appropriate in larger glands and those with medical comorbidities increasing the risk of bleeding, such as those on anticoagulation. Prospective studies are necessary to confirm these benefits and identify the select patients that will benefit from this approach.

## 5. Conclusions

Water-jet ablation has emerged as an effective treatment for BPH with its respective technology similar to TURP. Pre-operative PAE can reduce bleeding-related complications associated with water-jet ablation alone in select patients.

## Figures and Tables

**Table 1:** Summary of patient demographics and treatment variables before propensity score matching.

	Before Propensity Score Matching		
	Aqua	PAE + Aqua	
	(n = 56)	(n = 89)	*p* Value
Age, mean (SD)	62.2 (8.6)	69.6 (8.9)	<0.001
PRE-TREATMENT VARIABLES			
PSA, mean (SD)	3.1 (5.3)	4.2 (3.1)	0.292
Qmax (mL), mean (SD)	10.1 (5.3)	9.5 (6.3)	0.624
PVR (mL), mean (SD)	113.2 (111.2)	140.7 (125.5)	0.241
IPSS, mean (SD)	18.1 (7.4)	16.0 (8.8)	0.247
QOL, mean (SD)	3.6 (1.4)	3.8 (4.1)	0.802
IIEF, mean (SD)	16.1 (6.9)	15.9 (6.8)	0.901
Urocuff Peak Flow, mean (SD)	10.6 (8.7)	9.5 (4.6)	0.549
Urocuff Detrusor Pressure, mean (SD)	120.4 (46.9)	142.5 (50.8)	0.102
Prostate Gland Size (mL), mean (SD)	52.6 (24.8)	79.0 (27.3)	<0.001
INTRA-/PERI-TREATMENT VARIABLES			
Number of Passes Required for Treatment, mean (SD)	1.8 (0.5)	2.0 (0.2)	<0.001
Case Length (minutes), mean (SD)	58.7 (22.7)	57.4 (16.7)	0.722
Hospital Length of Stay (hours), mean (SD)	35.2 (24.1)	18.3 (19.1)	<0.001

**Table 2:** Summary of patient demographics and treatment variables after propensity score matching.

	After Propensity Score Matching		
	Aqua	PAE + Aqua	
	(n = 56)	(n = 89)	*p* Value
Age, mean (SD)	62.2 (8.6)	60.5 (109.6)	0.912
PRE-TREATMENT VARIABLES			
PSA, mean (SD)	3.2 (5.5)	3.6 (13.8)	0.877
Qmax (mL), mean (SD)	10.1 (5.4)	13.4 (33.4)	0.519
PVR (mL), mean (SD)	116.8 (112.7)	144.5 (339.3)	0.613
IPSS, mean (SD)	18.00 (7.5)	14.6 (24.7)	0.446
QOL, mean (SD)	3.6 (1.4)	3.2 (5.1)	0.704
IIEF, mean (SD)	15.8 (6.8)	19.7 (47.7)	0.685
Urocuff Peak Flow, mean (SD)	10.7 (8.8)	14.7 (41.1)	0.631
Urocuff Detrusor Pressure, mean (SD)	118.1 (46.4)	200.0 (498.8)	0.429
Prostate Gland Size (mL), mean (SD)	52.3 (24.7)	55.6 (96.7)	0.803
INTRA-/PERI-TREATMENT VARIABLES			
Number of Passes Required for Treatment, mean (SD)	1.8 (0.5)	2.1 (4.2)	0.631
Case Length (minutes), mean (SD)	58.2 (22.7)	53.9 (109.6)	0.789
Hospital Length of Stay (hours), mean (SD)	34.4 (23.5)	24.7 (75.3)	0.347

**Table 3:** Odds ratio analysis of adverse events. * Aquablation alone: PAE + aquablation.

	After Propensity Score Matching	
	Odds Ratio *	*p* Value
Discharged with a Foley catheter	0.3	0.004
Continuous Bladder Irrigation Required > 2 h	7.6	<0.001
Hemostatic Measures Required	14.7	<0.001
Hematuria	3.0	0.013
Urinary Retention	17.1	0.021
Dysuria	2.8	0.227
Bladder Irrigation Required	2.7	0.146
Required reoperation within 30 days	1.8	0.361
Required reoperation after 30 days	13.0	0.003
Required Foley catheter for greater than 30 days	8.7	0.233

## Data Availability

The original contributions presented in the study are included in the article, further inquiries can be directed to the corresponding author/s.
